# Factors enhancing implementation of occupational safety and health management systems in manufacturing industry of Mutare, Zimbabwe

**DOI:** 10.3389/fpubh.2025.1450567

**Published:** 2025-01-29

**Authors:** Johanes Mandowa, Mark Matsa, Steven Jerie

**Affiliations:** Department of Geography, Environmental Sustainability and Resilience Building, Midlands State University, Gweru, Zimbabwe

**Keywords:** sustainability, occupational safety and health management system, occupational safety and health, preventive safety culture, contextual factors, Mutare manufacturing industry, occupational safety and health management systems implementation, factors

## Abstract

**Introduction:**

Several studies elucidating a plethora of factors that influence establishment of OSH management systems in many industrial sectors have been conducted worldwide. Very few studies have been conducted to explicate factors influencing OSHMS implementation in Southern African countries, particularly in Zimbabwe. The study evaluated factors enhancing implementation of OSH management systems in the manufacturing industry of Mutare.

**Methods:**

A descriptive cross-sectional research design was utilized in the study. Primary data sources were questionnaires and interviews, and secondary data sources were e-OSH databases and past OSH research papers among others.

**Results:**

The results revealed the primary factors that enhance OSHMSs implementation as strong senior management commitment, involvement and support, strong employee involvement and participation, good safety culture, provision of adequate resources among others. Strong senior management commitment, involvement and support was identified as a catalyst of all other factors enhancing implementation of OSHMSs, hence a recommendation is made for employers to invest in programmes aimed at bolstering management commitment, involvement and support to OSH management.

**Discussion:**

Considering that lack of adequate resources emerged as a significant impediment to OSHMS implementation, the study challenges the Government to take a leading role in establishing policies that unlocks various funding mechanisms to support workplaces in overcoming financial barriers in the implementation of OSHMSs. It can be inferred from the study results that employee involvement is central to effective implementation of OSHMSs as it enables the generation of knowledge, ideas and deployment of abilities that are paramount in enhancing OSH management. Inferential statistics also revealed the existence of an association between factory size and implementation of OSHMS. Development of scaled down OSHMSs requirements for small to medium manufacturing companies (SMMCs) is recommended considering the unattractiveness of OSHMSs in SMMCs owing to sustainability challenges. This recommendation places a demand on researchers and OSH stakeholders globally to rethink the current global order for OSHMSs implementation in order to advance an approach to OSHMSs implementation in the SMMCs that is sensitive to contextual compounding factors. The study develops a framework that is key in creating a “preventive safety culture” at workplaces which is an epitome for sustainability in OSHMSs implementation.

## 1 Introduction

ILO (1) estimates that on an annual basis an average 2.3 million workers die from occupational injuries and diseases corresponding to over 6,000 deaths every single day. These figures are not just statistics but represent an unpleasant global Occupational Safety and Health (OSH) performance that is responsible for great human loss and suffering. Concerns about the debilitating effects of OHS failure to the future of work birthed the realization of the irreplaceable importance of Occupational Safety and Health Management Systems (OSHMSs) in the equation to attain business sustainability (2, 3). The application of safety systems in the current work dispensation has increasingly gained traction as a promising strategy to harmonize OSH and business sustainability (3, 4, 94). The ILO Centenary Declaration for the future of Work adopted in June 2019 declared a systems approach to OSH management as an essential fundamental for decent work and securing the future of work. Several authors assert that the concept of sustainability in development becomes untrustworthy if it is not entangled with the prevention of occupational safety and health risks (5–7). Many studies into OSHMSs have demonstrated that implementation of an OHSMS breeds progressive positive impacts on corporate security performance, economic and financial performance, and corporate competitiveness (2, 3, 8). Despite the revealed benefits of OSH management systems’ implementation and their widespread promotion globally, uptake of OSHMSs has regrettably remained low at many workplaces in Africa (9, 10, 94).

Several studies elucidated several factors that influence either negatively or positively the establishment of OSH management systems in many industrial sectors (11–13). Studies conducted in Saudi Arabia, South Africa, Honduras, India, Hong Kong, Kuwait, Uganda, China, and Jordan, highlighted factors that affect implementation of OSH management systems that include extensive subcontracting, inadequate safety training, absence of safety officers on site, ineffective laws and lack of enforcement, lack of workers’ self-protection and awareness, inadequate work procedures, poor accident record-keeping and lack of management commitment to safety budget (14–16). A study conducted by Tejamaya et al. (17) revealed many negative factors that lead to lack of interest and motivation in OSHMSs implementation in many micro, small to medium enterprises (MSMEs) that include among others the perception that OSH risks are low in MSMEs, short term benefits that are not clear and not outweighing the huge initial investment costs involved. Authors (18–21) are agreed on lack of human resources, ineffective information and communication, low financial support and low advisory services as chief factors responsible for the subdued uptake of OSHMSs in MSMEs. There is general consensus amongst many researchers that key factors that influence the effective implementation of OSH management systems include the following: provision of adequate resources (17, 19–21), management commitment and support (11, 22, 23, 95), employee involvement (22–24), good safety culture (25, 26), training (11), allocation of authority and responsibility (27, 28), the need to comply with OSH legislative provisions (29, 30), institutional image and reputation (31, 32) and improved productivity and profitability (33–35).

The majority of studies that have been conducted globally to explore the factors that enhance OSHMSs implementation (17, 19–21) were focusing on workplaces in developed countries that have a different contextual environment to that of developing and least developed countries. Andowa et al. (94) study demonstrated lower implementation levels of OSHMSs in most developing and least developed countries than in developed countries which may suggest the existence of variation in the contextual factors driving implementation of OSHMSs between the workplaces in developing and developed countries. Andowa et al. (94) study cautioned about the risk of applying the ‘one size fits all approach’ to OSH management system implementation at workplaces without considering the peculiar compounding factors that are a direct result of the disparities in the political, socio-economic environmental context. The study placed a challenge on researchers to conduct further research that will explore the factors associated with implementation of OSH management systems contextualized to the environmental setting of developing and least developed countries thereby providing the basis for establishment of problem driven solutions to increase uptake of OSH management systems at workplaces.

It cannot therefore be ruled out that failure to have a thorough understanding of contextual factors that enhance OSH management systems implementation is an impediment to effective promotion of OSH management systems at workplaces. Against an assertion by Pritchett et al. (99) that advocates for problem driven solutions adaptable to the environmental context, it is imperative to have a deeper understanding of the contextual factors enhancing OSHMSs implementation so as to craft problem driven solutions to increase uptake of OSHMSs.

Few studies have been conducted to explicate factors that drive OSHMS implementation in Southern Africa (36, 37, 94). This research gap in Southern Africa precipitates an unsustainable situation where most workplaces in developing and least developed countries are constantly resorting to taking the risk of experiencing failure in OSH management systems implementation by duplicating OSH management system implementation interventions from studies conducted largely in developed countries that are not necessarily compatible with their local environmental conditions. Zimbabwe, like any other country in Southern Africa is not spared from occupational safety and health challenges confronting the generality of workplaces. According to Mutetwa (38), Zimbabwe has been suffering from economic loss of approximately US$44 million annually due to occupational injuries and diseases. A sector analysis of the occupational accidents statistics done by NSSA singled out the manufacturing sector in Zimbabwe as the second most contributor to the economic loss Zimbabwe is suffering coming after the mining sector with a contribution of 0,39% of the Gross Domestic Product (GDP) (38). NSSA is convinced that the bad OSH performance of the country is mainly influenced by non-implementation of OSHMSs at many workplaces in the country as evidenced by the low annual national average uptake rate of OSHMSs of 13% recorded over a period of ten years from 2010 to 2020 (38–40). This low uptake rate of OSHMSs in Zimbabwe is worrisome as it is achieved against concerted efforts by NSSA to promote implementation of OSHMSs at all workplaces in Zimbabwe (38, 41).

Mutare manufacturing industry is therefore not an exception to a myriad of problems affecting implementation of OSH management systems in many workplaces in Zimbabwe (NSSA OSH Division Report for 2021). In 2021, Mutare experienced an upsurge of 11, 4% in occupational accidents as compared to the same period in 2020 despite the subdued economic activity in Zimbabwe owing to the effects of COVID 19 pandemic (NSSA OSH Division Report for 2021). Mandowa (42) and Jerie (97) noted that the bad OSH performance associated with Mutare is largely influenced by the dominance of the highly hazardous timber based manufacturing factories that are generally characterized by heavy, dirty and dangerous work requiring un-ergonomic working postures and exposures to noise, dust and vibration. This unpleasant performance is a cause for concern as it is not reflective of the overall OSH burden owing to unaccounted statistics of the informal sector which according to Rodas and Alas (43) accounts for three quarters of Zimbabwe’s working population. Mandowa (42) study fumigated non implementation of OSHMSs in timber processing factories of Mutare as militating against successful safety and health management. It’s a reality that the continued entertainment of such a negative OSH performance trajectory by Mutare manufacturing industry has the propensity to curtail the decent work and sustainable development agendas for the country.

The research question that remains an unexplored dilemma is why Mutare manufacturing industry which is known for its hazardous trait and the propensity to cause serious occupational accidents remain seemingly reluctant to embrace a systems approach to OSH management yet according to Kineber et al. (44), the benefits of OSHMSs implementation were proven as far outweighing the costs of OSHMSs implementation. Currently, literature has shown that there is no research that has been conducted specifically to explore factors that enhance implementation of OSH management systems in the manufacturing industry of Mutare yet it is an industry that is progressively contributing towards the bad OSH performance of Zimbabwe. An understanding of the factors that enhance OSHMS implementation was therefore necessary in the development of a framework for promoting the factors that enhance implementation of OSHMSs at workplaces taking into consideration several limitations that confront small, medium and large enterprises such as low budget, limited technical knowledge, and shortage of specialized labour (18, 45).

The study sought to contribute to the body of knowledge by challenging the current knowledge on OSH management systems implementation in developing countries and generate new ideas and information that are critical in strengthening the business case for a systems approach to OSH management not only in the manufacturing industry of Mutare but in many industrial sectors globally that are a duplication of the Zimbabwe’s political, socio-economic environmental context. The objectives of the study were therefore to assess the factors enhancing implementation of OSH management systems in the manufacturing industry of Mutare and to develop a framework for propagating the factors that enhance OSH management systems implementation at enterprise level Against such a background, the study therefore sought to answer the following research questions

- What are the contextual factors enhancing implementation of OSHMSs in manufacturing industry of Mutare?
- What framework is ideal to promote the factors that enhance OSHMSs implementation in the Manufacturing industry of Mutare.

## 2 Materials and methods

### 2.1 Study area

**Figure 1:**
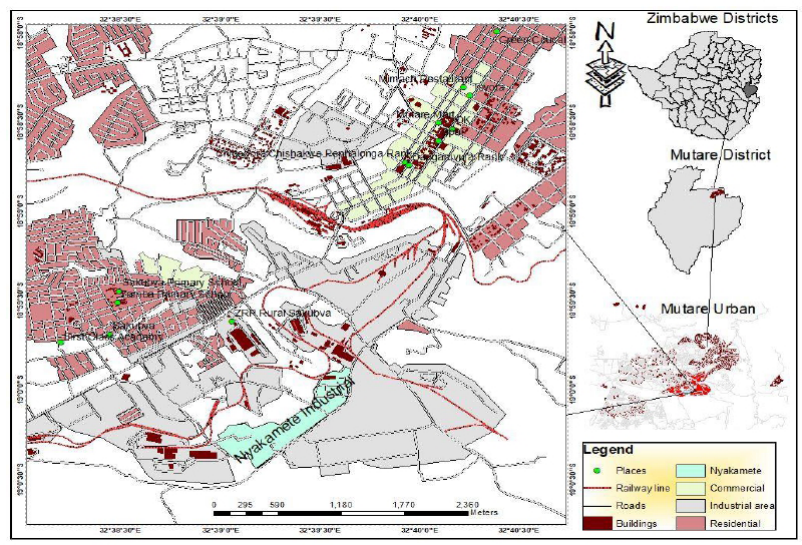
Map of the city of Mutare. Source: researchers.

City of Mutare (Figure 1) is the capital of Manicaland Province and the fourth largest city in Zimbabwe. According to Manatsa (46), City of Mutare was founded in 1897 as a fort, about 8 km from the border with Mozambique to the East. The Zimbabwe 2022 census revealed that Mutare city council has a population of 224,802 of which 106,602 are males and 118,200 are females (47). Mutare earned the pseudonyms “Zimbabwe’s Gateway to the Sea” and ‘Gateway to Eastern Highlands’ because of its proximity to Mozambique’s Beira port and the Eastern Highlands, respectively (97). The city’s proximity to the sea makes it the most strategic location for import and export-oriented enterprises (48).

The economy of Mutare is anchored by various business sectors that include forestry, agriculture, mining, tourism, manufacturing and informal sectors supported by a range of commercial and social services (49). Mutare economy is largely driven by manufacturing industry that is mainly located in the Nyakamete industrial area. Mutare city’s manufacturing industry is comprised of timber processing, food manufacturing and processing, engineering and other manufacturing factories. The manufacturing industry of Mutare is generally known for its hazardous trait (42) owing to the dominant timber based manufacturing factories that according to Jerie (96) are characterized by a host of occupational hazards that include among others engineering hazards of unguarded dangerous moving parts of machinery, physical hazards (such as exposure to excessive noise, dust and vibration) and ergonomic hazards (such as poor work postures, visual strain, poor workstation design). Most of the working population in Mutare is therefore formally employed in the timber based manufacturing factories, hence is increasingly vulnerable to occupational accidents and diseases owing to its exposure to highly hazardous work activities that characterize the generality of the dominant timber based manufacturing factories.

### 2.2 Method

King (50) regards a research design as a systematic procedure for data extraction, analysis, interpretation and reporting in research studies. Dube et al. (51), posits the need for a deep understanding of the philosophical underpinnings of the study to inform the study design. Relativism ontological approach which provides an understanding that adopts situation based evaluations rather than using absolute principles (52) was found ideal for this study based on a firm understanding that the reality about the factors that enhance OSH management systems’ implementation is subjective and will vary from person to person and from one organization to another. According to Hampson and McKinley (53), epistemological assumptions are concerned about knowledge gathering, creation and developing of new knowledge through a cocktail of methods of knowing. Perception experience (empiricism) and inference (rationalism) epistemological assumptions were adopted to acquire knowledge through perception experience and inference about the reality of the factors enhancing the implementation of OSHMSs in manufacturing industry of Mutare.

The underlying ontological and epistemological assumptions informed the methodological approach for this study of a descriptive cross-sectional study anchored on a mixed method research embracing both the quantitative and qualitative research paradigms. As noted by Cherry (54), a descriptive cross sectional study method provides the researcher with an opportunity to explore the conditions and potentially related characteristics of a given population at a specific point in time thereby allowing the researcher to extract data that is reflective of what is currently prevailing in the manufacturing industry of Mutare as far as the factors enhancing implementation of OSH management systems are concerned. According to Dube et al. (51), pragmatism is a pluralistic and practical approach which is viewed as the primary philosophical framework of mixed method research. Prasad (55) observed that pragmatism approach is handy to researchers in various fields, because of its inclination towards delivering results that are of practical significance. The choice of the mixed method study design was motivated by its ability to triangulate divergent viewpoints, opinions, perspectives, and standpoints (56) on the factors that enhance implementation of OSH management systems in Mutare manufacturing industry. Triangulation of the data was important in enhancing the veracity of the research findings thereby aiding the extrapolation of meaningful conclusions that generalize the factors enhancing implementation of OSH management systems in manufacturing industry of Mutare. Jafer et al. (57) affirmed the criticality of data triangulation as an enabler in the generation of data that gives the researcher more confidence about the validity of the research findings.

### 2.3 Population, sample size and methods of sampling

The study population consisted of 1,356 employees drawn from 30 manufacturing factories of Mutare that were registered by NSSA factories inspectorate as at 15 April 2022 and seven key OSH stakeholders that included Ministry of Public Service, Labour and Social Welfare (MPSLSW), The Academia (Mutare Poly Technical College), Employers’ Confederation of Zimbabwe (EMCOZ), Zimbabwe Congress of Trade Union (ZCTU), National Social Security Authority (NSSA), International Labour Organization (ILO) and Standards Association of Zimbabwe (SAZ). The rationale for the choice of key OSH stakeholders is as depicted in Table 1.

**Table 1:** Key OSH stakeholders and rationale for their selection.

Name of stakeholder	Targeted personnel	Rationale for selection
1. Ministry of Public Service, Labour, and Social Welfare (MPSLSW)	1. Provincial Labour Officer for Manicaland	The MPSLSW articulates the Government of Zimbabwe’s position on OSH administration in the country. The Provincial Labour Officer represents the MPSLSW on OSH issues in Mutare, hence was handy in articulating the Government views as far as the factors that enhance OSH management systems implementation are concerned. Furthermore, the MPSLSW is responsible for spearheading the enactment of labour laws such as the envisaged OSH bill hence the involvement of the Provincial Labour Officer helped to ensure that the Government of Zimbabwe is fully sensitised on factors that enhance OSHMSs implementation at workplaces which could be handy in triggering labour laws reformation.
	2. Labour Officer for Manicaland	Labour Officer for Manicaland Province is responsible for conducting labour inspections in the entire province, hence was poised to have experience-based knowledge on specific factors enhancing OSH management system implementation in the manufacturing industry of Mutare.
2. The Academia (Mutare polytechnical college)	Head of Engineering Department	The Engineering department advance OSH in various technical programmes offered by the college to support the manufacturing industry of Mutare hence the Head of Engineering helped in providing information that was useful in understanding the complexion of the factors that influence OSHMSs implementation in Mutare manufacturing industry
3. Employers’ Confederation of Zimbabwe (EMCOZ)	EMCOZ Representative in Mutare	EMCOZ represents the employers’ interests in OSH as OSH sustainability is critical for business profitability and survival. The EMCOZ Representative for Manicaland oversees EMCOZ interests in industries of Manicaland, hence was expected to be abreast with information on the factors contributing to improved implementation of OSH management systems in manufacturing industry of Mutare.
4. Zimbabwe Congress of Trade Union	Safety and Health Officer	The ZCTU is an employee’s body championing the cause of employees at workplaces in Zimbabwe. The ZCTU Safety and Health Officer is the focal person for OSH issues, hence was expected to be handy in proffering employee perspective about factors enhancing OSH implementation in the manufacturing industry of Mutare
5. NSSA	1. Director of Occupational Safety and Health	The Director of OSH is the custodian of OSH in Zimbabwe hence his involvement was critical in proffering NSSA’s perspective on factors enhancing implementation of OSH management systems in the manufacturing industry of Mutare
	2. Occupational Safety Health Promotions and Training Officer (OSHPTO)	The OSHPTO is the one responsible for spearheading the promotion of safety systems in industry hence was expected to be pregnant with information on some of the factors enhancing the adoption of OSH management systems at workplaces, Mutare manufacturing industry included
	3. Principal Inspector of Factories for Mutare	The Principal Inspector of Factories is the Chief inspector’s designate responsible for the effective enforcement of the OSH laws in all industries in Manicaland Province hence was ideal in highlighting in-depth information on factors enhancing OSHMSs implementation specifically in the manufacturing industry of Mutare
6. Standard Association of Zimbabwe (SAZ)	Standards Manager for Manicaland	SAZ’s mandate is to facilitate the formulation of OSH management systems standards, such as ISO 45001 and encouraging their implementation. The Standards Manager for Manicaland is responsible for this portfolio and was handy in proffering SAZ position on factors enhancing implementation of OSHMSs in manufacturing industry of Mutare
7. ILO	ILO Country Designate	ILO among other deliverables provides technical expertise to capacitate workplaces on OSH management systems’ implementation and their involvement will enable the extraction of technical expertise on factors enhancing implementation of OSHMSs and what needs to be done to ensure improved implementation of OSHMSs.

According to the NSSA factories classification system, factories are stratified as follows: A (Large~100 and above employees), B (Medium~51–99 employees) and C (Small~1–50 employees) as shown in Table 2.

**Table 2:** Distribution of manufacturing companies according to the NSSA factories classification system.

Class	Number of manufacturing companies				Total
	Timber based manufacturing	Food manufacturing	Engineering	Other manufacturing	
A: *N* = 100 and above	3	4	0	0	7
B: *N* = 51–99	2	0	0	0	2
C: *N* = 1–50	9	4	5	3	21
Total	14	8	5	3	30

Source: field data. NB: *N = Number of employees in the class.

A sample size of 309 employees was determined by applying the Slovin’s formula at an error margin of 0.05 according to the formula below;

$n=N/\left[1+N{\left(e\right)}^{2}\right]$

Where;

*n*: sample size.

*N*: proportion of the population.

*e*: margin of error = 0.05.

*n* = 1,356/[1 + 1,356 (0.05)^2^]

*n* = 309

The sample size was 22.79% of the study population thereby providing confidence on its representativeness of the sample as it was more than 10% generally considered by many researchers as good enough sample size to authenticate the research findings. Each manufacturing factory was anonymized by being given a unique reference code (identifier) that consisted of a unique number and two distinct letters, one representing nature of manufacturing business (letters T, F, E and O representing Timber based, Food manufacturing, Engineering and Other Manufacturing respectively) and the other representing class of the manufacturing factory [A (large ~100 and above employees), B (medium~51–99 employees) and C (small~l-50 employees)] respectively. Anonymization of the identity of the factory was paramount to uphold confidentiality of participating companies and their representatives so as to offset possible risks of victimization and institutional reputational damage. The sample size was proportionately distributed (Table 3) to all the 30 manufacturing factories in Mutare manufacturing industry according to the formula (58):

**Table 3:** Proportion of sample sizes as determined by Slovin’s formula.

Nature of manufacturing business	Company classification/code									Population	Prop of sample size
	Class A (100 and above workers)			Class B (51–99 workers)			Class C (1–50 workers)				
	Code	No of workers	Sample Size	Code	No of workers	Sample size	Code	No of workers	Sample size		
Timber based	TA1	120	27	TB4	51	12	TC6	35	8		
	TA2	150	34	TB5	52	12	TC7	12	3		
	TA3	100	23				TC8	20	5		
							TC9	10	2		
							TC10	30	7		
							TC11	5	1		
							TC12	28	6		
							TC13	16	4		
							TC14	11	3		
Total		**370**	**84**		**103**	**24**		**167**	**39**		
Food manufacturing	FA15	100	23				FC19	10	2		
	FA16	143	33				FC20	32	7		
	FA17	180	41				FC21	7	2		
	FA18	123	28				FC22	16	4		
Total		**546**	**125**					**65**	**15**		
Engineering							EC23	12	3		
							EC24	9	2		
							EC25	7	2		
							EC26	16	4		
							EC27	11	3		
Total								**55**	**14**		
Other manufacturing							OC28	40	9		
							OC29	4	1		
							OC30	6	1		
Total								**50**	**11**		
Grand total		**916**	**209**		**103**	**24**		**337**	**79**	**1,356**	**312**

Source: field data. NB, Prop of Sample Size means proportion of sample size. Prop of Sample Size means proportion of sample size. No means number. Letters T, F, E and O on company code depict nature of manufacturing business, Timber based, food manufacturing, engineering and other manufacturing, respectively, letters ABC on the code depict class of company according to the NSSA factory classification system; A representing company in class A category of 100 and above workers, B representing company in class B category of 51–99 workers and C representing company in class category of 1–50 workers. The sample size for each company were rounded off to the nearest ten; hence the 312 sample size projected on this table is more than the Slovin’s calculated sample size of 309. The values in bold in Table 3 represent the total number of workers and sample size for each nature of business.

$n_{1}=\frac{X_{n\;}\;x\;309}{\mathrm{N = 1,356}}$

Where;

n_1_ = Proportion of sample size in a particular manufacturing factory.

X_n_ = Number of the workers in a particular manufacturing factory.

N = Total target population in the manufacturing industry of Mutare.

Simple random sampling technique that entailed application of random number tables was used to sample the 309 worker respondents for questionnaire administration. A rotary method was utilized to select the sample for worker respondents from the manufacturing industry of Mutare. A semi-structured questionnaire was chosen to extract data from workers owing to its ability to collect comparable data as well as to ensure achievement of anonymity and confidentiality which was paramount in boosting the questionnaire response rate. The simple random sampling technique was chosen owing to its ability to ensure equal probability of selection for each worker respondent in the sampling frame thereby enhancing the representativeness of the sample (59) which is critical in aiding generalizability of the findings in the manufacturing industry of Mutare. More importantly simple random sampling technique was ideal to enable the application of inferential statistics to draw meaningful conclusions that generalized the characteristics of the population. Involvement of workers as respondents was critical as it resonated well with observations by several authors that worker participation is an irreplaceable ingredient in successful implementation of OSHMSs (60–62). Stratified random sampling technique was used to draw 10% of the workers from the questionnaire respondents (total of 309 employees) for further data elucidation through follow up interviews basing on the acceptability of 10% of the study population as sufficient sample size in a survey (63, 64). The distribution of the number of interviewees from the worker constituency is tabulated in Table 4.

**Table 4:** Distribution of the number of interviewees from the worker constituency.

Class	Number of worker interviewees				Total
	Timber based manufacturing	Food manufacturing	Engineering	Other manufacturing	
A: *N* = 100 and above	8	14	0	0	**22**
B: *N* = 51–99	2	0	0	0	**2**
C: *N* = 1–50	3	1	1	1	**6**
Total	**13**	**15**	**1**	**1**	**30**

Source: Field data. NB: *N* = number of employees. The values in bold in Table 4 represent the total number of employee interviewees per each class of business and for each type of manufacturing factory.

Questionnaire administration was also purposively extended to 30 and 11 respondents from the Top manager and Safety and Health Officer’s categories, respectively. This was necessitated by the need to collect comparable data from Top managers and Safety and Health Officers that would answer the research objectives.

Stratified purposive sampling technique was used to select 10% apiece of the Safety and Health Officers and Top managers for further in-depth data elucidation through interview administration. A Safety and Health Officer and a Top manager were targeted as interviewees because they are critical in OSHMSs implementation, hence were expected to be privy to factors that influence the implementation of safety systems. Purposive sampling technique was deployed to select one informant from each of the identified key OSH stakeholders (NSSA, EMCOZ, ZCTU, MPLSW, SAZ, ILO and Academia) for further in-depth interviews. Purposive sampling is a non-probability sampling technique that allows the researcher to use personal judgment to select the study respondents based on the basis of the knowledge of the population, its elements and the nature of your research aims (65). Purposive or judgmental sampling informed the selection of safety and health officers, top managers and key OSH stakeholders owing to the need for the researcher to extract specific in depth data that best answered the research objectives. Cronbach’s alpha (100) was applied on the questionnaires to assess their reliability. An acceptable reliability coefficient of 0.85 was found considering the generally acceptable Cronbach alpha reliability coefficient of 0.70 and above in most research situations (66).

**Figure 2:**
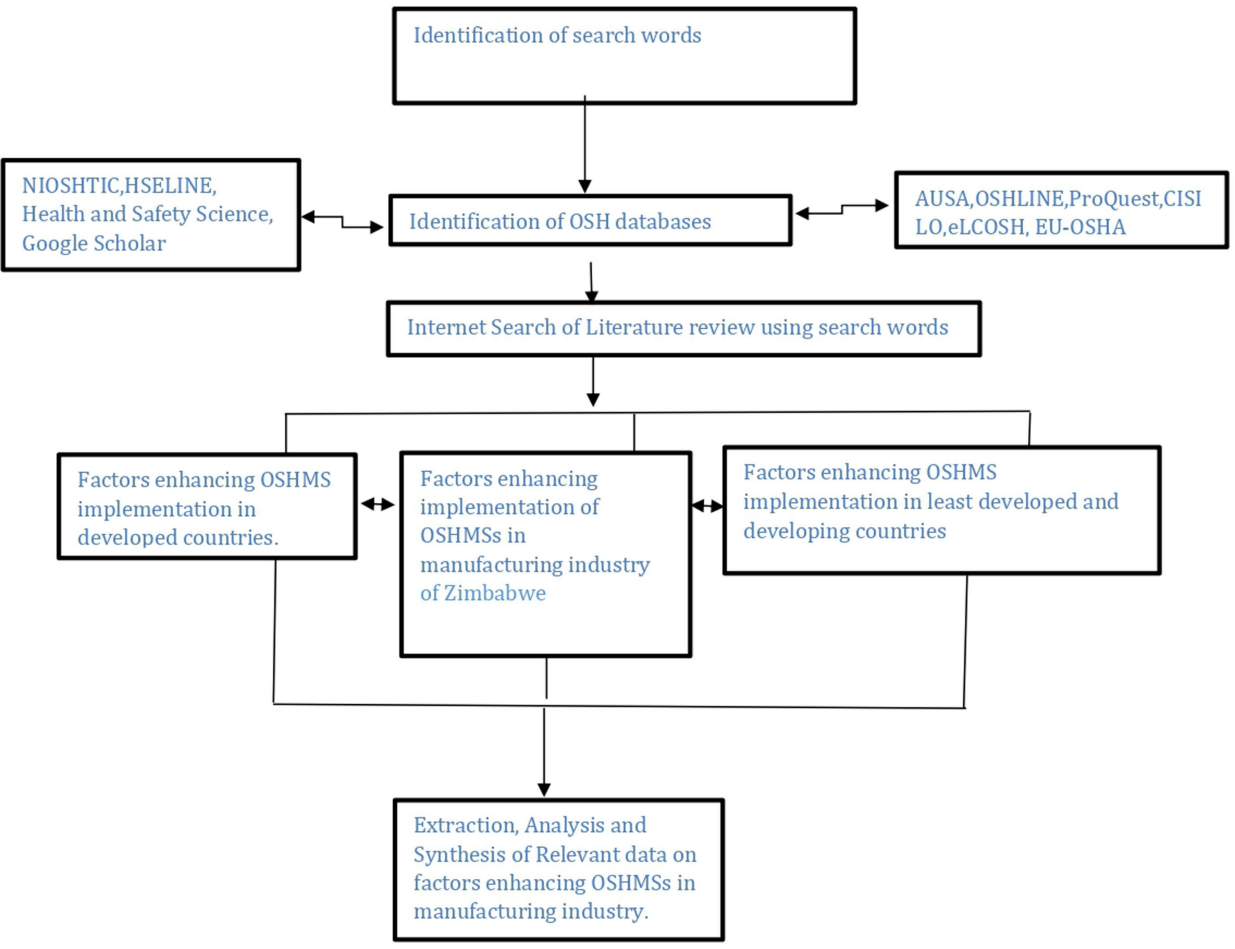
Schematic representation of secondary data search strategy. Source: researchers.

Secondary data was extracted from past OSH research papers, ILO OSH conventions, OSH policy and legislation in Zimbabwe, OSH encyclopedia and other relevant OSH textbooks, OSH journals and NSSA’s records on factory inspection, registration and occupational injuries statistics among other sources. Key guiding words and phrases for internet search were established with the objectives of the research in mind. The key guiding words or phrases utilized included among others; Challenges of implementing OSHMSs, OSHMSs implementation in developing countries/developed countries, factors influencing OSHMSs implementation, employee involvement in safety management, safety culture in OSH management, management commitment in OSHMSs and safety training and awareness in OSH management. Figure 2 is a schematic representation of the strategy for secondary data internet search.

### 2.4 Variables for analysis

#### 2.4.1 Factory size

Several authors have confirmed factory size as a proxy variable for implementation of OSHMSs (11, 12, 22, 23, 67, 96). Based on this understanding, the study explored a hypothesis to establish whether there was an association between factory size and implementation of OSHMSs in the context of the manufacturing industry of Mutare.

Hypothesis 1.

H_0_: There is no association between factory size and the implementation of OSH Management System.

H_1_: There is association between factory size and the implementation of OSH Management System.

According to Nordlöf et al. (67), a company size is normally measured by either the company’s turnover or the number of employees. A more universal measure of the number of employees was adopted to determine the factory size for each manufacturing company. The NSSA factories registration database that captures factory sizes was utilized to establish the number of employees at each manufacturing factory. Furthermore, the number of workers in each factory was validated by data provided by Human Resources departments of respective companies.

#### 2.4.2 Employee involvement

Employee involvement in OSH was found to be strongly linked to employee commitment in OSH management (17, 60). Based on this observation by Tejamaya et al. (17) and ISSA (60), the study made a hypothesis to establish whether there was an association between employee involvement and implementation of OSHMSs in the context of the manufacturing industry of Mutare.

Hypothesis 2.

H_0_: There is no association between worker involvement and the implementation of OSH Management System.

H_1_: There is association between worker involvement and the implementation of OSH Management System.

An instrument that generally measures employee involvement cannot be found in literature (98); hence the researcher was motivated to design this measure by considering all best practices for employee involvement as revealed in literature. The following 9 factors were therefore adopted and used in this study to portray the state of employee involvement in OSH management in the manufacturing industry of Mutare: evaluation of PPE, establishment of OSH policy, Safety and Health committee, safety communication, safety audits and inspections, safety promotional programmes (safety talks, competitions, safety drills), risk assessment, incident and accident reporting, incident, and accident investigation. Questions and statements aligned to the 9 parameters for measuring employee involvement in OSH management in the manufacturing industry of Mutare were formulated and answered using a Likert scale with 5 options (Table 5) acknowledged by McLeod (68) as handy in allowing the respondents to indicate their positive to negative strength of agreement regarding a given question or statement.

**Table 5:** Likert scale.

Strongly disagree	Disagree	Undecided	Agree	Strongly agree
(1)	(2)	(3)	(4)	(5)

### 2.5 Data analysis and presentation

#### 2.5.1 Descriptive and inferential statistics

Descriptive statistics were grouped into defined data sets according to the research objectives, analyzed, presented in statistical manner using frequent tables, and pie charts and then discussed continuously in a descriptive manner. In terms of inferential statistics, the independent variables in this study were factory size and employee involvement and the dependent variable was implementation of OSH management system. Responses to questions that contained the variables of interest were coded to ease the application of Statistical Package for Social Science (SPSS) software, version 16 in the determination of association between variables of interest. Chi Square test of independence was performed at 5% significance level to test whether there was an association between independent variables (factory size and employee involvement) and implementation of OSH management system (dependent variable). The choice of the Chi square test was informed by the fact that both variables (independent and dependent) were categorical and the data to be analysed was from a random sample. Responses for the number of employees in a factory (factory size) were coded as reflected in Table 6.

**Table 6:** Factory size survey coding sample.

Number of employees	Code
1–50	1
51–99	2
100 and above	3

Each of the 9 primary factors that measure employee involvement was coded as follows; Strongly Agree and Agree were coded 1(Yes) and Strongly Disagree, Disagree and Undecided were coded 0 (no). The items were summed together to create that index (scores 0–9) as predictor variable in the main analysis. SPSS software was used to perform a Chi Square test of independence at 5% significance level to establish the existence or nonexistence of an association between independent variables (factory size, employee involvement) and implementation of OSH management system (dependent variable) thereby enabling some inference to be drawn about the characteristics of the population.

SPPS software was used to present the results of the Chi square test of independence in the form of cross tabulations showing the observed and expected counts, Pearson Chi-Square test result and Cramer’s V and Phi coefficient results. The *p*-value, or Asymptotic Significance of the Pearson Chi – square test was used to determine the existence of a statistically significant relationship between the variables of interest. The *p*-value that was less than 0.05, resulted in the null hypothesis being rejected and a conclusion being made that there is a statistically significant relationship between the variables. The opposite was true for *p*-value greater than 0.05.

Cramer’s V and Phi coefficient are vital measures for assessing the strength of association between two categorical variables (69). The two measures were applied to assess the strength of association between independent variables (factory size, employee involvement) and dependent variable (implementation of OSH management system) with a normalized value between 0 and 1. The Phi and Cramer’s V results were interpreted in accordance with Table 7.

**Table 7:** Interpretation of Phi and Cramer’s V.

Phi and Cramer’s V	Interpretation
>0.25	Very strong
>0.15	Strong
>0.10	Moderate
>0.05	Weak
>0	No or very weak

Source: Akoglu (93).

#### 2.5.2 Interview and secondary data analysis and presentation

Data from interviews was extracted from the written notes and recorded audios and compiled according to the research objectives. A comparative analysis was done to synthesize and group the data into defined data sets according to similarities and differences. Based on the research objectives, similar thoughts from the interview data were quantified and tabulated as exemplified in Table 8.

**Table 8:** Interview sample findings.

Data set 1	
Objective: factors that enhance OSH management systems implementation	Number of informants
Strong senior management commitment	5
Provision of adequate resources	10
Strong employee involvement and participation	15

Source: field data.

Significant differences of thoughts from the interview data were noted. The interview data was presented in a descriptive manner in the results and discussion sections to augment other data sources. Secondary data was synthesized and reviewed in a descriptive manner to augment other data sources.

### 2.6 Ethical considerations

Ethical conduct has increasingly received greater attention globally in the field of research in response to society’s expectation of greater accountability (70). Respondents’ consent was sought prior to the commencement of the data collection exercise through the administration of a consent form that was voluntarily signed independently by both the respondents and participating manufacturing factories without any form of coercion. Participating respondents and manufacturing factories were anonymized in line with the Committee of Publishing Ethics (71) assertion on the need to avert the potential risks of irreparable reputational institutional damage and victimization of research participants. World Health Organization (WHO) Covid 19 guideline (72) was implemented to guarantee a strict adherence to covid 19 measures that included sanitization, utilization of face masks, maintenance of social distancing and temperature screening. Highest level of professionalism, beneficence, justice, confidentiality and personal integrity (73) was exercised during questionnaire and interview administration so as to ensure that the respondents understood the purpose of the study and were comfortable throughout the entire research process. Bailey (74) lamented the risk of misinterpretation and misrepresentation of facts in the process of gathering data, hence the researcher granted the respondents an opportunity to validate the representativeness of the research paper before it was submitted for publishing.

## 3 Results

The results section covers the questionnaire and interview response rates, an exploration of the factors enhancing implementation of OSHMSs in manufacturing industry of Mutare, and development of a framework to promote the factors that enhance OSHMSs implementation in the Manufacturing industry of Mutare.

### 3.1 Response rate

**Figure 3:**
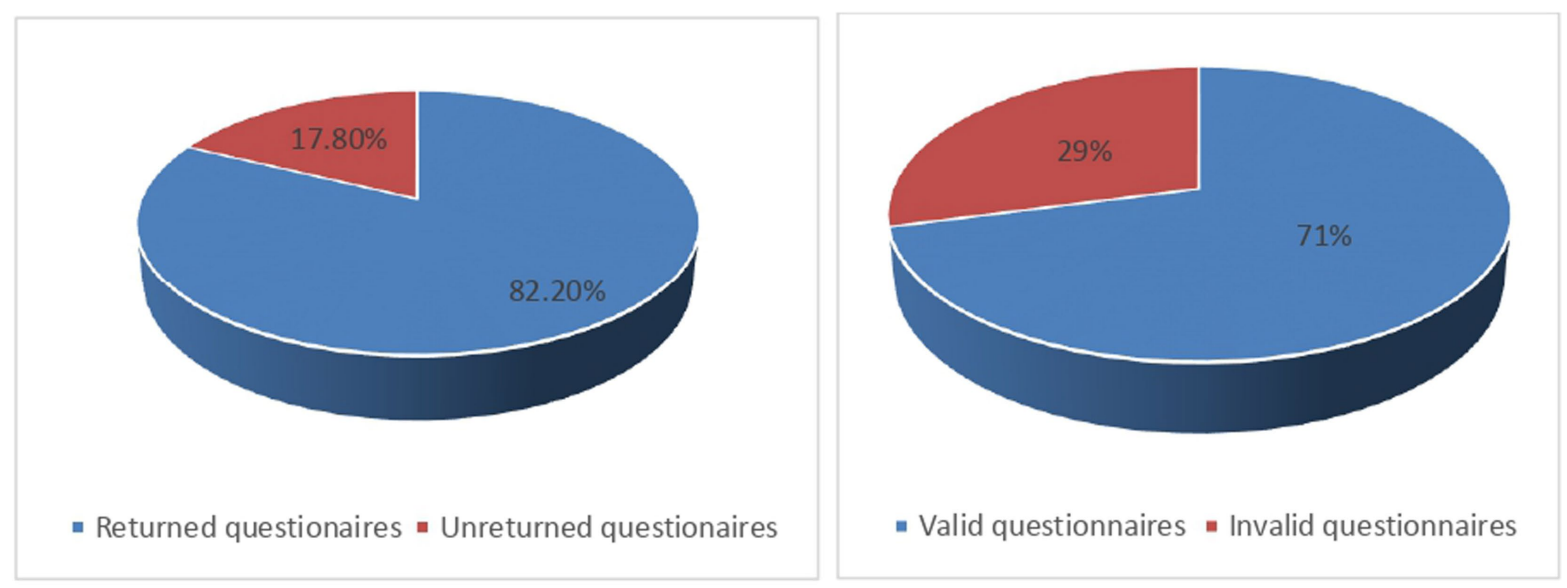
Worker questionnaire response rate.

A worker questionnaire response rate of 82.2% was achieved as a total of 254 questionnaires were successfully returned for analysis out of the anticipated 309 questionnaires (Figure 3).

Analyses for questionnaire validity produced 180 (70.9%) valid questionnaires. One hundred percent (100%) and 87.1% of questionnaires distributed to OSH practitioners and Top managers, respectively, were valid for data analysis. All (100%) of the targeted interviewees (workers, Safety and Health Officers, Top managers, and key OSH stakeholders) were successfully interviewed. Response rates for both questionnaires and interviews were good enough to guarantee the authenticity and validity of the research findings taking cognizance of Njogu et al. (11) proclamation of the acceptability and validity of a response rate of 70% and above in a study.

### 3.2 Factors enhancing implementation of OSHMSs in manufacturing industry of Mutare

As reflected in Table 9, the generality of the respondents agreed that strong senior management commitment, involvement and support (89%), strong employee involvement and participation (91%), good safety culture (93%), provision of adequate resources (89%) and training of all personnel on the importance of OSH management systems (90%) were the top five factors enhancing implementation of OSH management systems in manufacturing industry of Mutare.

**Table 9:** Factors enhancing implementation of OSHMSs in manufacturing industry of Mutare.

Factors enhancing OSH management system implementation		Workers	Key OSH stakeholders	Safety and health officers	Top managers	Average score
		%	%	%	%	%
1	Strong senior management commitment, involvement, and support	83.3	93.8	100	78	89
2	Provision of adequate resources	77.8	93.8	100	85	89
3	Making OSH performance a component of performance appraisal for management and workers	80.6	93.8	90	89	88
4	Strong employee involvement and participation	85.5	93.8	100	85	91
5	Good safety culture	84.4	93.8	100	93	93
6	Training of all personnel on the importance of OSH management systems	81.2	100	90	89	90
7	Mandatory law for OSHMS implementation	78.4	87,5	80	89	84
8	Need to comply with OSH legislative provisions	82.3	81.3	80	85	82
9	Need to protect institutional image and reputation	82.2	75	80	74	78
10	Improved productivity and profitability	77.2	81.3	70	81	77
11	Incentives in the form of reduced Workers Compensation Insurance Fund’s premiums	64.4	81.3	70	63	70
12	Punitive penalties for non-compliance to OSH legal requirements	65	68.8	90	52	69
13	Markets that demand OSHMS as a condition for trade	68.9	93.8	80	56	75
14	Availability of platforms for mutual learning and networking	70.6	87.5	80	59	74

Application of Chi square test at 5% significance level with 1 degree of freedom to test the existence of an association between employee involvement and implementation of OSHMSs revealed a P_value_ of 0.001 which was less than the 0.05 significance level (Chi square test results shown in Tables 10, 11). As the *p*-value was less than the 0.05 significance level, the null hypothesis (H_0_) was rejected and the alternative hypothesis (H_1_) was accepted. A conclusion was drawn from the test result that there was an association between employee involvement and implementation of OSHMS implementation.

**Table 10:** Employee involvement * OSHMS Implementation Cross tabulation.

			OSHMS implementation		Total
			OSHMS not implemented	OSHMS implemented	
Employee involvement	Disagree	Count	78	0	78
		Expected count	56.3	21.7	78.0
	Agree	Count	52	50	102
		Expected count	73.7	28.3	102.0
Total		Count	130	50	180
		Expected count	130.0	50.0	180.0

**Table 11:** Pearson Chi-Square tests for association between employee involvement and OSHMS implementation.

	Value	df	Asymp. sig. (2-sided)	Exact sig. (2-sided)	Exact sig. (1-sided)
Pearson Chi-Square	52.941^a^	1	0.000		
Continuity correction^b^	50.526	1	0.000		
Likelihood ratio	71.340	1	0.000		
Fisher’s exact test				0.000	0.000
Linear-by-Linear association	52.647	1	0.000		
N of valid cases^b^	180				

a 0 cells (0.0%) have expected count less than 5. The minimum expected count is 21.67.

b Computed only for a 2×2 table.

Application of Phi and Cramer’s V to assess the strength of association between employee involvement and implementation of OSH management systems revealed a Phi and Cramer’s V value of 0.542 (Table 12) which was greater than 0.25 thereby implying the existence of a very strong relationship between employee involvement and implementation of OSHMSs.

**Table 12:** Phi and Cramer’s V symmetric measures for strength between employee involvement and OSHMS implementation.

		Value	Approx. sig.
Nominal by nominal	Phi	0.542	0.000
	Cramer’s V	0.542	0.000
*N* of valid cases		180	

The Chi square result therefore confirmed the strong view by most respondents (91%) that strong employee involvement and participation was a key factor in enhancing implementation of OSHMSs. The majority of key stakeholder interviewees’ (93.8%) and safety and health officers (100%) concurred with the overall opinion of the generality of the respondents on the top five factors enhancing implementation of OSHMSs. Furthermore, all the key OSH stakeholders were agreed that training of all personnel on the importance of OSH management systems and how to implement them was a key enhancing factor to OSHMSs implementation as it had a bearing on the manifestation of other factors. Most of the Top managers (90%) interviewed corroborated the general view by key OSH stakeholders by asserting the criticality of harnessing knowledge on OSHMSs through training; however, they lamented lack of adequate human and financial resources as an impediment to execution of the trainings especially in organizations categorized as SMEs (small to medium enterprises). A categorical analysis revealed that training of workers on OSH was more pronounced in Timber based (76.9%) and food manufacturing (61.8%) companies than in engineering (42.9%) and other manufacturing (0%) companies. Furthermore, the results revealed that most of the employees that received occupational safety and health training after joining their respective organizations were working in Timber based and Food manufacturing organizations that had an appointed safety and health officer and that had implemented an OSHMS.

A significant number of respondents (84%) as reflected in Table 9 cited the need for a mandatory law for OSHMSs implementation as a factor that enhances implementation of OSHMSs in the manufacturing industry of Mutare. Augmenting this view, NSSA interviewees asserted the importance of having a robust OSH legislation that captures the minimum requirements for an OSHMS as a tool to compel organizations to take up OSHMSs. However, NSSA bemoaned the low levels of OSH enforcement at workplaces owing to inadequate provision of operational resources as a drawback to realization of positive results even in the event that the legislation is reformed to legalize establishment of OSHMSs. This bottleneck of low levels of OSH enforcement was echoed by most workers (58, 3%) and key OSH stakeholders (62, 5%) that rated OSH enforcement in the country to be below satisfactory. Majority of the workers rated their organization’s overall compliance to OSH legislation as poor (61, 1%) whilst others rated their organization’s OSH compliance as satisfactory (32.2%), good (3.9%) and very good (2.8%).

Provision of adequate resources was highlighted as a factor enhancing implementation of OSHMSs by 77.8% of the workers and 89% of all the research respondents. It is interesting to note from the results that most of the worker respondents in all the categories of the manufacturing industry of Mutare that concurred on the provision of adequate resources as an enhancing factor to OSHMSs implementation described their remuneration as poor [timber based (37.4%), food manufacturing (43.4%), engineering (42.9%) and other manufacturing (66.7%)]. ZCTU acknowledged the importance of provision of adequate resources as a factor propelling the implementation of OSHMSs, however, it emphasized on the need to buttress this factor with other factors considering the available evidence in literature that attests to unavailability of OSHMSs even in organizations with healthy financial resources. Ninety three-percent (93%) of the respondents were generally convinced that a good safety culture in an organization was a central factor that enhances the implementation of OSH management systems in the manufacturing industry of Mutare. ILO asserted the need for organizations to build an organizational safety culture as a progressive step towards effective implementation of OSHMSs. NSSA interviewees cited good safety culture as a key factor that is a derivative of the availability of all other factors that enhance OSHMSs implementation.

#### 3.2.1 A stratified analysis of the factors enhancing the implementation of OSHMSs in manufacturing industry of Mutare

A stratified analysis of the factors that enhance the implementation of OSHMSs is revealed in Table 13.

**Table 13:** Stratified analysis of factors enhancing implementation of OSHMSs in the manufacturing industry of Mutare.

Factors enhancing OSH management system implementation		Timber based	Food manufacturing	Engineering	Other manufacturing
		%	%	%	%
1	Strong senior management commitment, involvement, and support	75	83	85	90
2	Provision of adequate resources	64	84	85	66
3	Making OSH performance a component of performance appraisal for management and workers	69	80	71	67
4	Strong employee involvement and participation	72	85	71	83
5	Good safety culture	71	89	86	100
6	Training of all personnel on the importance of OSH management systems	70	88	71	100
7	Mandatory law for OSHMS implementation	69	73	71	100
8	Need to comply with OSH legislative provisions	69	80	72	100
9	Need to protect institutional image and reputation	67	89	71	100
10	Improved productivity and profitability	61	85	71	83
11	Incentives in the form of reduced Workers Compensation Insurance Fund’s Premiums	53	68	71	33
12	Punitive penalties for non-compliance to OSH legal requirements	53	73	43	66
13	Markets that demand OSHMS as a condition for trade	53	75	58	83
14	Availability of platforms for mutual learning and networking	69	75	71	66

**Figure 4:**
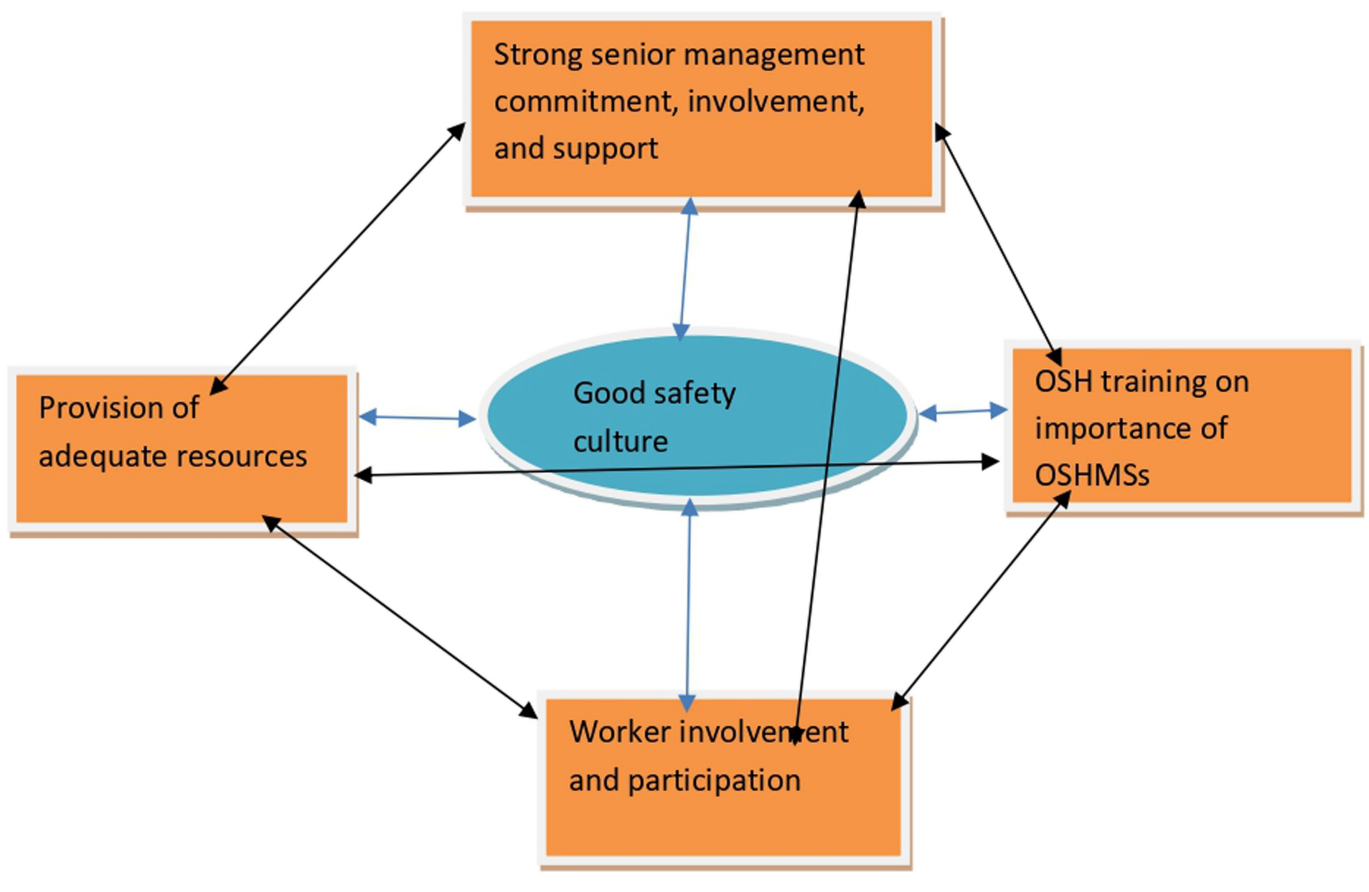
Schematic representation of the interdependence existing amongst factors enhancing implementation of OSHMSs. Source: researchers.

The stratified analysis of all the categories of companies making up the manufacturing industry of Mutare revealed a duplication of the same trend that strong senior management commitment, involvement, and support, provision of adequate resources, strong employee involvement and participation, good safety culture and training of all personnel on the importance of OSH management systems are the dominant factors enhancing implementation of OSHMSs. This revelation tends to suggest that the factors that enhance implementation of OSHMSs equally influence all the manufacturing factories irrespective of the nature of business in which the manufacturing company is involved. From the analysis in Table 13, a good safety culture emerged as the most influential factor enhancing OSHMSs implementation in all the four categories of companies that make up the manufacturing industry of Mutare. According to Tejamaya (17), a good safety culture in an organization reflects the manifestation of interdependent factors enhancing implementation of OSHMSs that include senior management commitment, worker involvement and participation, provision of adequate resources among others. Considering the assertion by Tejamaya (17), this interdependence can be demonstrated as depicted in Figure 4.

The results from Top manager questionnaire respondents revealed that most of the manufacturing factories (66.7%) making up the Mutare manufacturing industry served the local market only whilst 33.3% served both local and international markets. A stratified analysis of the manufacturing factories as differentiated by the number of employees according to the NSSA factories classification system showed that all medium (51–99 employees) and big (100 and above employees) factories (100%) served both local and international markets whilst 72% of small factories (1–50 employees) concentrated on the local market only with a paltry 28% of the small factories supplying both local and international markets. It is interesting to note that all the top five enhancing factors to OSHMSs implementation were more evident in medium and big factories (100 employees and above) that served both the local and international markets than in small factories that served the local markets only. In tandem with this observation, application of Pearson Chi square test of independence at 5% significance level with 2 degrees of freedom to test the existence of an association between factory size and implementation of OSHMSs revealed a P_value_ of 0.000 which was less than the 0.05 significance level. Tables 14, 15 show factory size and OSHMSs implementation cross tabulation and the Pearson Chi square test results after application of SPSS version 16 software.

**Table 14:** Factory size * OSHMSs implementation cross tabulation.

			OSHMSs implementation		Total
			Not implemented	Implemented	
Factory size	Small	Count	19	2	21
		Expected count	15.4	5.6	21.0
	Medium	Count	2	0	2
		Expected count	1.5	0.5	2.0
	Large	Count	1	6	7
		Expected count	5.1	1.9	7.0
Total		Count	22	8	30
		Expected count	22.0	8.0	30.0

**Table 15:** Pearson Chi-square tests for association between factory size and OSHMS implementation.

	Value	df	Asymp. sig. (2-sided)
Pearson Chi-square	16.364^a^	2	0.000
Likelihood ratio	15.845	2	0.000
Linear-by-linear association	13.771	1	0.000
N of valid cases	30		

a 3 cells (50.0%) have expected count less than 5. The minimum expected count is 0.53.

As the *p*-value was less than the 0.05 significance level, the null hypothesis (H_0_) was rejected and the alternative hypothesis (H_1_) accepted. A conclusion was drawn from the test results that there was an association between factory size and implementation of OSHMSs.

Application of the Phi and Cramer’s V to assess the strength of association between factory size and implementation of OSH management system revealed a Phi and Cramer’s V value of 0.739 (as shown in Table 16) which was greater than 0.25 thereby implying the existence of a very strong relationship between factory size and implementation of OSHMSs.

**Table 16:** Phi and Cramer’s V symmetric measures for strength between factory size and OSHMS implementation.

		Value	Approx. sig.
Nominal by nominal	Phi	0.739	0.000
	Cramer’s V	0.739	0.000
N of valid cases		30	

### 3.3 Development of a framework to promote the factors that enhance OSHMSs implementation in the manufacturing industry of Mutare

The following framework was developed based on the interdependence and the mutually exclusivity of the factors that enhance implementation of OSHMSs in the manufacturing industry of Mutare.

**Figure 5:**
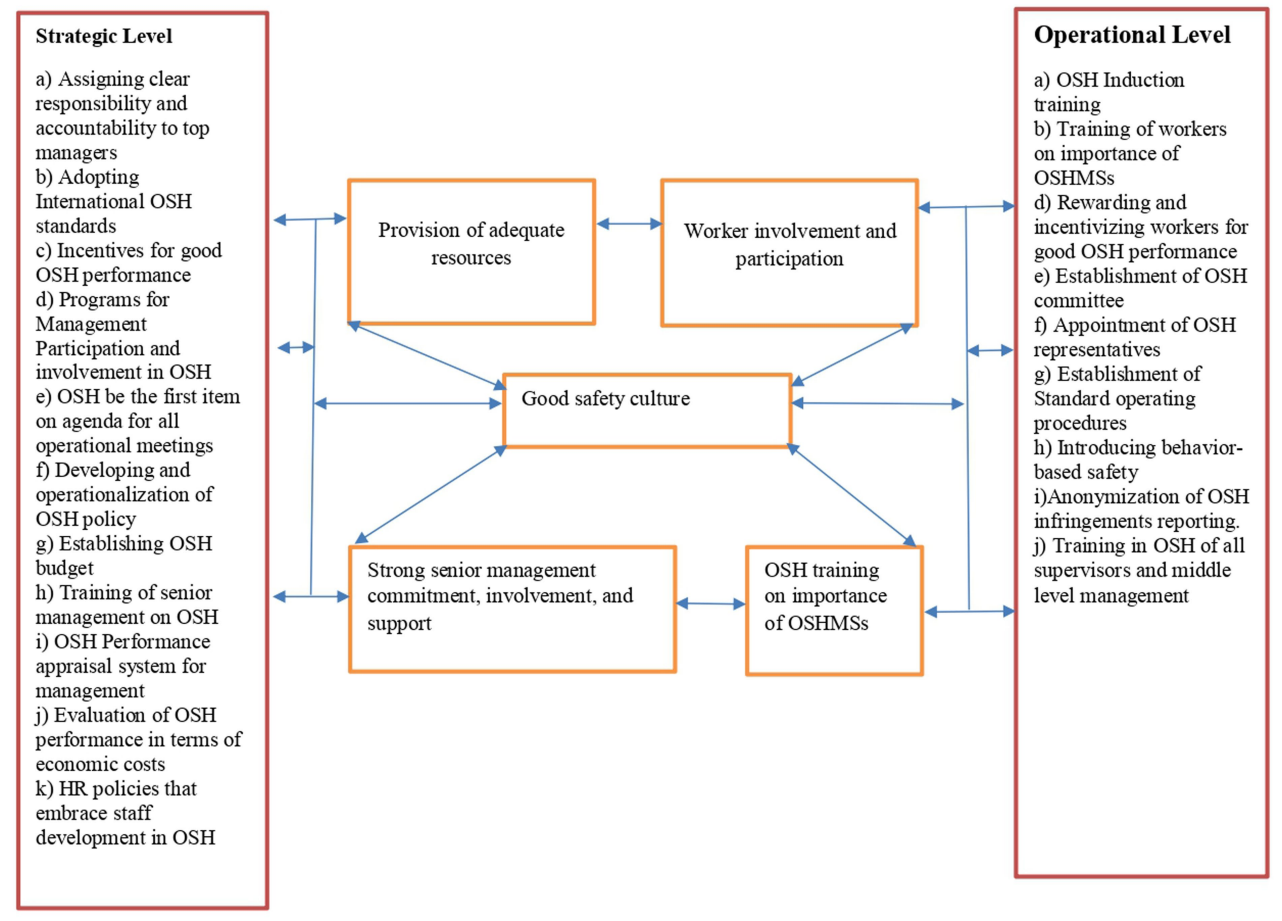
Framework for the promotion of factors that enhance OSHMSs implementation in the manufacturing industry of Mutare. Source: researchers.

As shown in Figure 5, the framework makes suggestions of several interventions that need to be taken on board at strategic and operational level to ensure the promotion of the factors that enhance implementation of OSHMSs in the manufacturing industry of Mutare. At strategic level the interventions are aimed at bolstering leadership and commitment which has a direct positive influence on the identified factors. The object of interest in OSH management is the worker, hence interventions at operational level are more worker centric as they seek to buttress the creation of good safety culture through worker participation and involvement. As reflected on the framework, strategic interventions such as formulation of the OSH policy that is compatible with the overall strategic objective and direction of the organisation, setting of OSH objectives and targets, establishing an OSH budget to unlocking resources, assigning clear responsibilities and accountability to all in positions of leadership, establishing HR policies that embrace OSH, establishing OSH training opportunities for top management, and ensuring OSH is the first item on the agenda in all operational meetings among others are key in promoting a good organizational safety culture that enhances OSHMSs implementation. The framework shows that at operational level, leadership has an obligation to invest in motivating employees’ participation and involvement by devising and rolling out programmes such as provision of awards and incentives for good OSH performance to encourage a healthy competition, establishing behavior based safety programme that ensures that workers are able to monitor each other’s behavior, appointment of OSH representatives, establishment of OSH committees and training of employees among others.

## 4 Discussion

### 4.1 Factors enhancing implementation of OSHMSs in manufacturing industry of Mutare

Several studies conducted globally over the years confirm the identified factors that enhance implementation of OSHMSs in the manufacturing industry of Mutare (11, 13, 17, 19, 28, 75–77). The revelation emerging out of this study that strong senior management commitment, involvement and support (89%) is one of the key factors enhancing implementation of OSHMSs corroborates findings of several studies conducted to explore the influence of management commitment to establishment of OSHMSs (17, 60, 75, 78). According to ISSA (60), top management commitment is the nucleus and the driving force behind all other factors (such as provision of adequate resources and training on importance of OSHMSs), that contribute to improved implementation of OSHMSs. It can be deduced that the concept: ‘commitment strategy for safety’ is one critical innovative perspective for the realization of a world of work without occupational accidents and diseases as it calls for long term commitment by top management to prevent all accidents thereby creating conditions in organizations where initiatives to improve safety are encouraged and can flourish.

Abdallah et al. (75) and Jitwasinkal et al. (78) identified the availability of a nexus between management commitment and employee commitment. In their studies, it was demonstrated that lack of management commitment has a negative impact on employee commitment further cementing long held ILO (101) view that ‘What management do not value cannot be valued by worker’. Management commitment as a factor can therefore be strengthened to support implementation of OSHMSs in manufacturing industry of Mutare through establishment and operationalization of an OSH policy, setting of OSH objectives and targets, establishing an OSH budget to unlock required resources, assigning clear responsibilities and accountability to all in leadership position, appointment of an OSH practitioner to coordinate OSH matters and reforming HR policies to embrace OSH and making OSH performance a component of performance appraisal for management among other measures.

Strong employee involvement and participation was cited by a significant number of respondents (91%) as another important factor enhancing implementation of OSHMS in manufacturing industry of Mutare. Application of Chi square test at 5% significance level to test the existence of an association between employee involvement and implementation of OSHMSs revealed the existence of an association between employee involvement and implementation of OSHMSs. The Phi and Cramer’s V value confirmed a very strong association between employee involvement and implementation of OSHMSs. These statistical results are in line with the fundamental value of the Vision Zero concept that places employees at the center of successful management of OSH (60). Employee involvement in OSH has been found to be strongly linked to employee commitment (22–24, 60) as this helps to foster a sense of ownership. The Vision Zero concept golden rule number 7 on ‘Invest in people; motivate by participation’ places the obligation on leadership to invest in motivating their employees by involving them in all safety and health matters. It therefore follows that when employees in manufacturing industry of Mutare are involved and consulted in critical safety programs such as risk assessment or in the development of operating instructions, their willingness to follow the rules is buttressed thereby aiding effective implementation of OSHMS. Ultimately, the importance of strong employee involvement and participation places a demand for manufacturing factories in Mutare to explore opportunities aimed at strengthening worker involvement and participation such as establishment of safety and health promotional programmes that include among others safety briefings, use of safety suggestion boxes, safety awareness days, OSH prizes and awards and safety competitions.

The largest number of respondents cited good safety culture (93%) as the top factor enhancing implementation of OSHMSs. Tejamaya (17) recognized a good safety culture in an organization as a reflection of the manifestation of interdependent factors enhancing implementation of OSHMSs that include senior management commitment, worker involvement and participation, provision of adequate resources among others. Creating a good safety culture should therefore be a strategic challenge for all manufacturing factories as it is a derivative of all other factors. It can be deduced from Figure 3 that factors that enhance implementation of OSHMSs are not mutually exclusive and effective implementation of the other factors contributes to positive achievement of a good safety culture where proactive safe behavior becomes the norm. Provision of adequate resources (86%) was also cited by a significant number of respondents as another important factor enhancing implementation of an OSHMS. Conversely, inadequate resources can lead to failure by an organization to provide the necessary resources to finance effective application of the hierarchy of risk control, hire qualified and competent OSH practitioners to spearhead the implementation of an OSHMS, provide OSHMSs training to both management and workers and to remunerate employees adequately. From the results, most of the worker respondents in all the categories making up the manufacturing industry of Mutare described their remuneration as poor further cementing the criticality of provision of adequate resources. The poor remuneration that characterizes the manufacturing industry of Mutare is worrisome as it is a psychosocial risk factor that has the propensity to increase the vulnerability of employees to occupational health risks and occupational accidents.

Eighty four percent (84%) of the respondents highlighted the need for a mandatory law for OSHMSs implementation as a factor that enhances implementation of OSHMSs in the manufacturing industry of Mutare. It is imperative to highlight that having legislation that make OSHMSs implementation mandatory at all workplaces will not necessarily translate into widespread compliance in the manufacturing industry of Mutare owing to inadequate capacity of NSSA to effectively enforce the legal provision arising out of limited human and material resources. According to Yangho (79) and ILO (102) there is a significant development in the OSH landscape where the command approach (prescriptive traditional OSH strategy) that proved to be unsuccessful and inefficient in inducing compliance since time immemorial is being overtaken by a goal setting philosophy of self-compliance arising from a global realization by organizations for the need to exercise their duty of care and responsibility for their workers taking cognizant of the sanctity of human life and the centrality of the human resource in attaining business productivity and sustainability.

Training of all personnel on the importance of OSH management systems (90%) emerged as one of the significant factors enhancing implementation of OSHMSs. Tejamaya (17) and Njogu (11) identified training as a critical factor aiding adoption and implementation of OSH Management systems. This assertion by the respondents resonates well with the Vision Zero Concept Golden rule number 6 on ‘Improve qualification-develop competences’ that recognizes training of employees in OSH management as a cog in ensuring the development of skills and competences necessary to attain zero harm (60). It is interesting to note from the categorical analysis that training of workers on OSH was more pronounced in Timber based (76.9%) and food manufacturing (61.8%) companies than in engineering (42.9%) and other manufacturing (0%) companies. This revelation can be explained by the fact that most of Timber based, and Food manufacturing organizations had appointed safety and health practitioners who influenced establishment of OSH training programmes considering their technical knowhow on the importance of OSH training in successful implementation of OSHMSs. There is therefore the need for development of OSH training strategies at both national and enterprise level to offset the gaps associated with suboptimal to nonexistent OSH training programmes at most workplaces making up the manufacturing industry of Mutare.

The revelation that all the top five enhancing factors to OSHMSs implementation were more evident in medium and big companies (100 employees and above) that served both the local and international markets than in small companies that served the local markets only is a pointer to the influence of the global markets in aiding OSHMSs implementation. As noted by Mandowa (42) global market forces are increasingly demanding sustainability in doing business thereby placing a demand on companies that export products to demonstrate beyond any reasonable doubt their commitment to safeguarding employees’ safety and health as a condition of doing business. Application of Chi square test at 5% significance level to test the existence of an association between factory size and implementation of OSHMSs revealed a P_value_ of 0.000 which was less than the 0.05 significance level thereby revealing the existence of an association between factory size and implementation of OSHMS. The Phi and Cramer’s V value of 0.739 confirmed a very strong association between factory size and implementation of OSHMSs. These statistical results implied that the attractiveness of OSHMSs is differentiated between big organizations and small organizations. As observed by Mandowa (42), large companies often have a large financial resource base that translate to increased capacity to implement OSHMSs. On the contrary, Micheli et al. (20) asserted lack of financial muscle on the part of small to medium businesses as a major contributory factor to the less commitment exhibited by these organizations in establishing OSHMSs. Tejamaya et al. (17) and Masi et al. (80) argued that OSHMSs are generally perceived as irrelevant in small to medium businesses (SMBs) owing to unavailability of a huge workforce to guarantee translation of OSHMS implementation into immediate direct monetary gain for the company thereby relegating OSHMSs as unimportant for companies’ survival. The arguments by Tejamaya et al. (17) and Masi et al. (80) can be compounded by lack of OSH expertise in small to medium scale enterprises to quantify OSH performance in terms of monetary gain or loss. It can be extrapolated that development of scaled down OSHMSs requirements for small to medium enterprises is ideal considering the huge financial resources that characterize implementation and sustenance of an OSHMS. A deduction can be made from the results that the criticality of promoting sustainability in OSH management cannot be underestimated. Taking from a globally accepted standpoint that a safe and health worker is more productive than an unsafe and unhealthy worker, it is implied that business productivity, profitability, and sustainable economic development are functions of a safe and healthy workforce. Failure to promote the realization of the factors enhancing OSHMSs implementation at every workplace making up the manufacturing industry of Mutare is a serious impediment not only to Zimbabwe’s quest to attain Agenda 2030 Sustainable Development Goal 8 on the promotion of Sustained, Inclusive and Sustainable Economic Growth, Full and Productive Employment and Decent Work for All but breeds some negative ripple effects on the country’s National Development Strategy (NDS1) (2021–2025) particularly on Health and Well-being, Social Protection, Environmental Protection and Economic Growth and Stability priority areas leading to curtailment of the country’s vision for an Upper Middle-Income Economy by 2030.

### 4.2 Development of a framework to promote factors that enhance OSHMSs implementation in the manufacturing industry of Mutare

Figure 4 demonstrated the importance for workplaces to take interventions at strategic and operational level targeted at boosting leadership and commitment and worker involvement and participation, respectively. ISSA (60) places leadership and commitment at the center of effective implementation of OSHMSs, hence it is critical for top management in the manufacturing industry of Mutare to lead OSH from the front by embracing all the suggested strategic interventions. Many researchers (17, 22–24, 60, 94) have asserted the criticality of employee participation, involvement, and commitment in fostering a prevention safety culture. It can therefore be extrapolated that measures aimed at ensuring full participation and involvement of all workers at a workplace enable the generation of knowledge, ideas and deployment of abilities that are paramount in enhancing the creation of a good safety culture taking cognizance of Garnica and Barriga (18) conviction that employees are better placed to follow safety rules when they are consulted.

## 5 Conclusion

It can be concluded from the study that the primary factors that enhance implementation of OSHMSs are strong senior management commitment, involvement and support, strong employee involvement and participation, good safety culture, provision of adequate resources, mandatory law for OSHMSs implementation and training of all personnel on the importance of OSH management systems. Strong senior management commitment, involvement and support is recognized as the driving force behind propelling all other factors enhancing implementation of OSHMSs (17, 60, 75, 78). This is vindicated by Abdallah et al. (75) and Jitwasinkal et al. (78) studies which identified the availability of a nexus between management commitment and employee commitment. It can be concluded that employers must invest in programmes aimed at improving management commitment to OSH as it has a direct positive impact on employee commitment as reinforced by ILO (101) assertion that what management do not value cannot be valued by workers.

Application of the Chi-square test of independence revealed the existence of an association between factory size and implementation of OSHMS thereby cementing the noticeable differences in uptake of OSHMSs between large companies and small companies. This observed association of factory size and implementation of OSHMSs resonates with Andowa et al. (94) study which revealed lower appetite in OSHMSs implementation in small to medium enterprises that in large enterprise. Studies by Hasle and Limborg (81) and Esterhuyzen (82) asserted unattractiveness of comprehensive OSHMSs in small companies as they are viewed as too complicated to be applied. Tejamaya et al. (17) confirmed smaller firms struggle more than bigger firms in terms of OSH management systems implementation. The observed nexus between the size of the enterprise and the provision of adequate resources is a direct result of the low capital levels that characterize the majority of the small to medium enterprises that make it difficult for them to implement and sustain a comprehensive OSHMS. Considering the huge financial demands associated with implementing comprehensive OSHMSs that cannot be sustained by the generality of the small to medium enterprises owing to low capital levels (20), the study recommends the development of an OSHMS framework with scaled down OSHMSs requirements that are ideal for the small to medium enterprises. This recommendation places a demand on researchers and OSH stakeholders globally to rethink the current global order for OSHMSs implementation that tend to advance a one size fits all approach of implementing off the shelf OSHMSs such as ISO 45001 standard without considering contextual compounding factors. The existence of an association between employee involvement and implementation of OSHMSs is a vindication of the centrality of workers in the equation for successful implementation of OSHMSs as revealed by many researchers globally. In congruency with evidence in literature that employee involvement in OSH is strongly linked to employee commitment (83–85), employers are challenged to devise programmes within the framework of OSHMSs implementation that enhance opportunities for employees involvement and participation. Full integration of all the factors that enhance implementation of OSHMSs at both national and enterprise level is critical considering that the enhancing factors are not mutually exclusive. Promotion of the factors enhancing OSHMSs implementation at every workplace should therefore be a strategic objective at both national and workplace level so as to ensure the attainment of OSH sustainability at all workplaces which is paramount to the attainment of the milestones under Agenda 2030, National Development Strategy (NDS 1) and the 2030 vision of an upper middle-income economy. The study cements the need for Manufacturing industry of Mutare to invest in creating a ‘preventive safety culture’ which is an innovative perspective of the Vision Zero concept advocating for a metamorphosis of workplaces from a pathological stage where there is ‘no care culture’ and no safety systems through to generative stage where OSHMSs become an epitome for successful management of OSH risks. The study develops a framework to promote propagation of the factors that enhance implementation of OSHMSs in the manufacturing industry of Mutare.

### 5.1 Recommendations

In line with the assertion by Vik-Benibo et al. (86) and ISSA (60) that leadership and commitment is the nucleus for effective implementation of OSHMSs, the study makes the following recommendations to promote implementation of OSHMSs:

1. Government should review the current OSH legislation to make it mandatory for workplaces to implement OSHMSs. The review of the OSH legislation should provide for minimum critical elements of an OSHMS that a workplace can implement taking into consideration the disparities in the implementation of OSHMSs between large and small to medium enterprises as observed by Mandowa et al. (2022) and Tejamaya et al. (17).
2. As noted by Micheli et al. (20) and Tejamaya et al. (17) that lack of financial muscle on the part of small to medium businesses is an impediment to OSHMSs implementation, the Government should establish various funding mechanisms through financial services sector to support the SMEs in overcoming financial barriers in the implementation of OSHMSs that include among others offering cheaper loan facilities for SMEs. Requirements to access the funding must incorporate the need for the organization to have implemented some minimum critical elements of an OSHMSs and this will induce implementation of OSHMS as businesses will not have a choice but to align with the requirement for them to be able to access funding.
3. The Government must review its procurement policy to incorporate safety and health requirements as one of the criteria for awarding all Government contracts. This policy position will induce implementation of OSHMSs as prospective contractors are required to satisfy this condition for them to be competitive in winning Government contracts.
4. NSSA, which is the competent authority in the management of OSH in the country should revolutionize its OSHMSs promotional strategy by adopting and operationalizing a risk-based approach aimed to offering long term technical assistance on OSHMSs implementation on an ongoing basis to organizations in a systematic and programmed manner guided by well-defined objectives and targets.
5. Employers are recommended to exhibit leadership and commitment in OSHMSs implementation by ensuring that.
  - Human resources policies at workplaces, for example recruitment policy (interview, medicals, conditions of services), training policy (induction, on job safety training, formal safety training, evaluation of the impact of training), Procurement and Tender awarding Policy, and Performance appraisal policy among others embrace some requirements for OSH to aid the creation a preventive safety culture which is key in the successful implementation of OSHMSs as observed by Tejamaya (17).
  - Employer representative bodies such as EMCOZ and (Zimbabwe National Chamber of Commerce) (ZNCC) should promote OSHMSs implementation by incorporating the need for workplaces to implement some critical elements of an OSHMSs as one of the criteria for evaluating businesses performance and individual performance of managers when determining winners for annual business awards: Such a requirement on the criteria for granting performance awards for businesses and managers will trigger healthy competition and motivate businesses and managers entrusted to run businesses to embrace OSH thereby contributing to a positive safety culture.
  - Employers must unlock resources necessary to support the implementation of OSHMSs by establishing an OSH budget which becomes part of the overall organizational annual budget. This will help to ensure that OSH is managed in a sustainable manner by availing the necessary resources to finance effective application of the hierarchy of risk control, hire qualified and competent OSH practitioners to spearhead the implementation of an OSHMS, provide OSHMSs training to both management and workers and to remunerate employees adequately, coming up with an workplace safety awards/incentive System for good OSH performance by employees that will induce healthy competition and motivate for a preventive safety culture as asserted by Caburao (103).
  - In line with research backed evidence that employee involvement in OSH is strongly linked to employee commitment (83–85), employers should create opportunities for employee involvement and participation in the management of OSH by coming out with programmes earmarked to enhance employee involvement and participation such as safety briefings or tool box meetings, OSH suggestion box, safety awareness days, prizes and awards system and safety competitions among others.

## 6 Limitations

Theofanidis and Antigoni (87) defines limitations of any particular study as potential weaknesses that are usually out of the researcher’s control, and are closely associated with the chosen research design, statistical model constraints, funding constraints, or other factors. A limitation on external validity of the study that affect the generalizability of the study was identified as emanating from the fact that the study concentrated on the manufacturing industry of Mutare thereby excluding other manufacturing factories in other regions of the country. However there was adequate confidence on the potential to generalise the research findings in manufacturing companies that are located in different geographical areas of the country and in other countries that are a duplication of the Zimbabwean socio-economic environmental context considering an assumption that the factors enhancing implementation of OSHMS could be similar. Future research should therefore strengthen this study to guarantee external validity by expanding the population to cover wide variety of workplaces in the manufacturing industry located in different geographic locations of the country. Another limitation was on the reproducibility of the results which could be compromised by inherent subjectivity of questionnaires and interviews. Miles and Scott (88) assert that interviews cannot be truly duplicated due to their subjectivity, hence cannot be reproduced over time. According to Kwapong (89) questionnaires and interviews have a limitation of a possibility of collecting data that is untruthful. Application of data triangulation technique that involved conducting follow up interviews to questionnaire administration and the synthesis of data from various sources was critical in countering the limitations of questionnaire and interviews. Another area of potential limitation was the data analysis methodology. As observed by Theofanidis and Antigoni (87), quantitative statistical analysis, such as Chi square test can easily determine statistical significance on the relationship between two or more variables, that does not necessarily imply that one variable has any causal effect on the other. A more detailed analysis would therefore be required in order to establish causality between the variables (87, 90–92).

## Data Availability

The original contributions presented in the study are included in the article/supplementary material, further inquiries can be directed to the corresponding author.
